# Regulation of Gap Junction Dynamics by UNC-44/ankyrin and UNC-33/CRMP through VAB-8 in *C*. *elegans* Neurons

**DOI:** 10.1371/journal.pgen.1005948

**Published:** 2016-03-25

**Authors:** Lingfeng Meng, Chia-hui Chen, Dong Yan

**Affiliations:** 1 Department of Molecular Genetics and Microbiology, Duke University Medical Center, Durham, North Carolina, United States of America; 2 Department of Neurobiology and Duke Institute for Brain Sciences, Duke University Medical Center, Durham, North Carolina, United States of America; The University of North Carolina at Chapel Hill, UNITED STATES

## Abstract

Gap junctions are present in both vertebrates and invertebrates from nematodes to mammals. Although the importance of gap junctions has been documented in many biological processes, the molecular mechanisms underlying gap junction dynamics remain unclear. Here, using the *C*. *elegans* PLM neurons as a model, we show that UNC-44/ankyrin acts upstream of UNC-33/CRMP in regulation of a potential kinesin VAB-8 to control gap junction dynamics, and loss-of-function in the UNC-44/UNC-33/VAB-8 pathway suppresses the turnover of gap junction channels. Therefore, we first show a signal pathway including ankyrin, CRMP, and kinesin in regulating gap junctions.

## Author Summary

The nervous system is made up of individual neurons connected by junction structures called synapses. There are two fundamentally different types of synapses: chemical synapses and electrical synapses (also called gap junctions). Through studies in different model organisms, we have gained rich knowledge about the development and dynamics of chemical synapses. However, we still know little about how gap junctions are formed during development and what regulates the dynamic of gap junctions in functional circuits. Using *C*. *elegans* PLM neurons as a model, we carried out an unbiased genetic screen and isolated mutants altering gap junctions. In this study, we focus on two of them, *unc-44/ankyrin* and *unc-33/CRMP*. Through genetic analysis in combination with live imaging, we find that UNC-44/ankyrin and UNC-33/CRMP play important roles in gap junction turnover and demonstrate that UNC-44/ankyrin acts upstream of UNC-33/CRMP and VAB-8/ kinesin to regulate the removal of gap junction channels from gap junctions.

## Introduction

Gap junctions were first discovered in the myocardium and nerves for their properties of electrical transmission between two adjacent cells [1,2], and they are clusters of channels connecting two cells to allow direct transfer of ions and small molecules [3–5]. Gap junctions play essential roles in many biological processes, such as embryo development, cell differentiation, cell growth, metabolic coordination of avascular organs, and neural development [6]. In excitable cells, the presence of gap junctions provides them with abilities to generate synchronized electrical and mechanical outputs [3–5]. Gap junction channels form polymorphic maculae or plaques with a few to thousands of units [4] and are composed of connexins in chordates and innexins in prechordates [7,8]. Although connexins and innexins are not homologs in terms of their primary sequences, they share similar structures with four transmembrane domains, two extracellular and one intracellular loop, and intracellular amino- and carboxy- termini [5]. Vertebrates have innexin-related proteins, called pannexins, however, their roles in forming gap junctions are still under debate [9,10].

Regulation of gap junctions is observed at two levels: fast regulation involving change of channel conduction and open probability, and slow regulation including alternation of composition and turnover of channels [4]. Studies show that voltage changes and phosphorylation of channel proteins are important for fast regulation of gap junctions [11–14]. In terms of slow regulation, the turnover of gap junctions plays an important role [15]. Gap junctions have remarkably rapid turnover rate, for example connexin/Cx 43 has a half-life of only 1–3 hours [16–20]. The rapid turnover rate allows cells to quickly eliminate and rebuild their gap junctions to adapt to environmental conditions [4]. Gap junction channel proteins can form gap junctions in homomeric or heteromeric manners, and different combinations have distinct permselectivity [4]. The rapid turnover rate of gap junctions provides cells with the ability to change the composition of gap junctions in a timely manner. During gap junction turnover, the addition of new channels is at the edge of gap junction plaques, and the removal of channels happens at the center of the plaques [21,22]. Although recent studies show that the phosphorylation of gap junction channels and channel binding proteins is involved in regulating gap junction turnover, the molecular mechanisms orchestrating the removal of gap junctions are still largely unknown [4].

Transient gap junctions are important for mammalian brain development [3,23–25]. In invertebrates, transient gap junctions can regulate the formation of chemical synapses in leeches [26] and are required for asymmetry development of sensory neurons in *C*. *elegans* [27]. To avoid interruption of neuronal functions, those transient gap junctions need to be eliminated during development, but it remains unknown what regulates their elimination. Understanding the molecular mechanisms underlying gap junction dynamics may answer this question.

As scaffolding proteins, ankyrins can organize membrane proteins into discrete domains and integrate them with the cytoskeleton [28]. In neurons, ankyrin-G is essential for the assembly of axon initial segment (AIS) and nodes of Ranvier [29,30] and is important for synapse formation [31,32]. Collapsin response mediator proteins (CRMP) are conserved microtubule interaction proteins that regulate neuronal polarity and axon growth [33–35]. *C*. *elegans* has only one ankyrin homolog *unc-44* and one CRMP homolog *unc-33*. Loss of function of *unc-44* and *unc-33* generate similar defects in locomotion, axon growth and axon-dendrite differentiation, suggesting they may function in the same pathway in regulating neuronal development [36–41]. Indeed, a recent study by Maniar et al. has shown that *unc-44* acts upstream and regulates the localization of *unc-33* in organization of microtubules in *C*. *elegans* neurons [41].

Although the important roles of ankyrin and CRMP in neuronal development have been documented in many organisms, their functions in regulating gap junctions have not been explored. Here, we show that in gap junction turnover, UNC-44/ankyrin acts upstream of UNC-33/CRMP and VAB-8/ kinesin to regulate the removal of UNC-9/innexin from gap junctions.

## Results

### Using *C*. *elegans* PLM neurons as a model to study gap junctions

**Fig 1:**
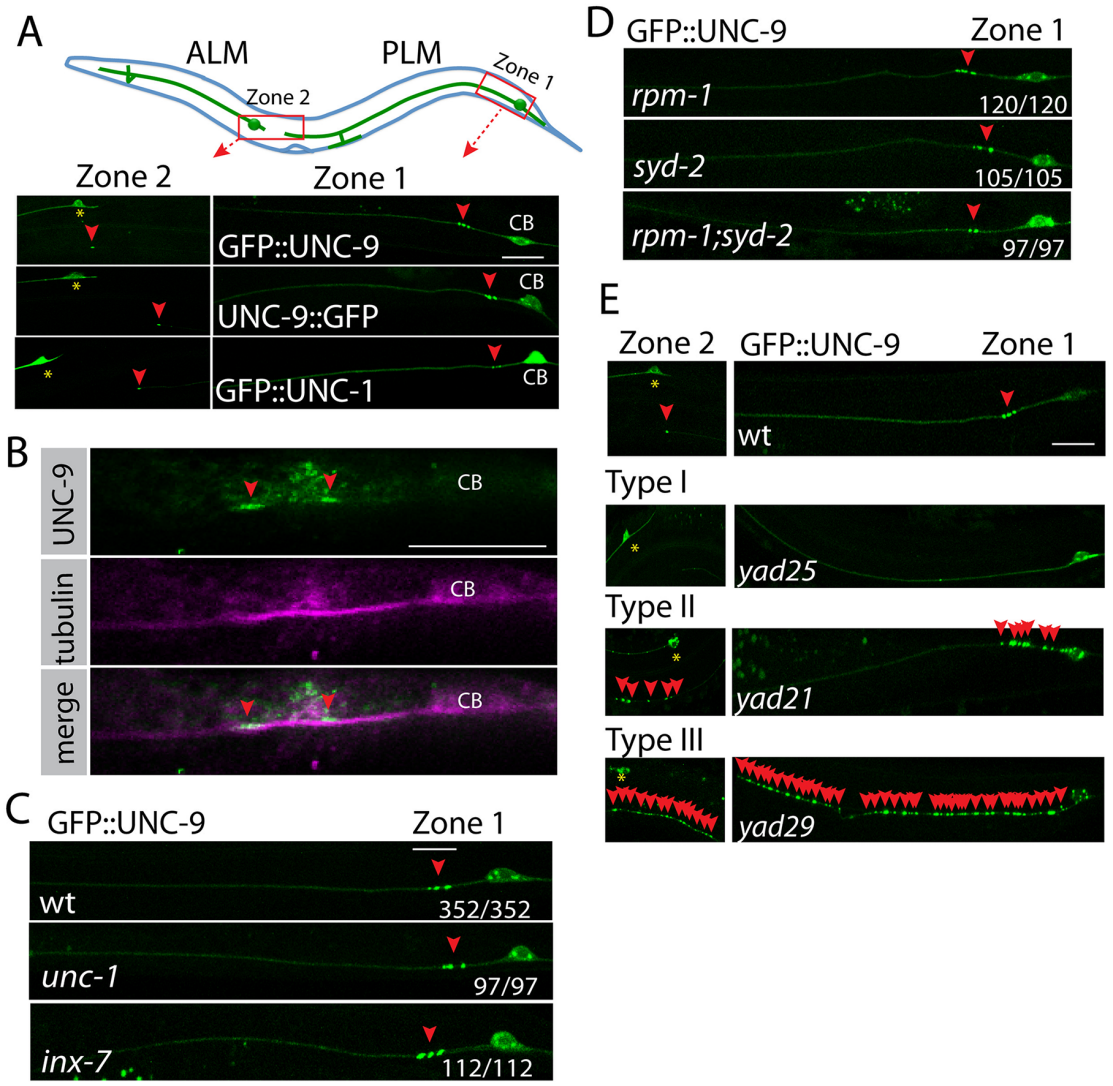
The cluster of UNC-9 puncta in PLM neurons represents gap junctions *in vivo*. **(A)** Expression patterns of GFP::UNC-9 fusion proteins represent the localization of gap junctions in PLM neurons. At the top panel, a cartoon picture shows the morphology of PLM neurons. The red rectangles highlight two gap junction zones in PLM neurons. In bottom panels, images of expression patterns of GFP::UNC-9, UNC-9::GFP and GFP::UNC-1 in PLM neurons. Red arrowheads point to GFP puncta at gap junction zones. Yellow stars label ALM cell bodies. CB: PLM cell bodies. **(B)** Images of immunostaining results show that endogenous UNC-9 forms similar punctate structures at gap junction zone 1. UNC-9 was stained using Rabbit anti-UNC-9 antibody (green color). PLM neurons were labeled using mouse anti-acetylated tubulin antibody (pink color). We confirmed those neurons were PLM neurons based on their morphology and the position of cell bodies. CB: PLM cell bodies. **(C)** Representative images show that loss-of-function mutations in *unc-1* or *inx*-7 does not affect GFP::UNC-9 puncta. **(D)** Suppression of chemical synapse formation by mutating *rpm-1* and *syd-2* does not affect GFP::UNC-9 puncta in PLM neurons. **(E)** Three types of mutants identified in the genetic screen for gap junctions. Red arrowheads point to GFP::UNC-9 puncta. Yellow stars label ALM cell bodies. In figures C and D, the number displayed at each image shows the number of animals with wild type GFP::UNC-9 puncta/ total animals. Scale bar: 10 μm. Detailed strain information of all figures is listed in the S1 Table.

To study molecular mechanisms underlying gap junction regulation, we used *C*. *elegans* PLM neurons as a model. PLM neurons are a pair of mechanosensory neurons with simple morphology, that cell bodies are located at the tail region with a long axon growing to the middle part of body and a short posterior process toward the end of tail (Fig 1A) [42]. Electron microscope studies showed that PLM formed gap junctions at two regions along the axon: at zone 1, PLM neurons form gap junctions with PVC, LUA and PVR neurons [43]; at zone 2, PLM neurons form gap junctions with BDU neurons [44] (Fig 1A). Three innexins, *unc-9*, *inx-7* and *inx-3*, are expressed in PLM neurons [45]. To visualize PLM gap junctions *in vivo*, we used GFP labeled UNC-9 as marker. UNC-9 has four transmembrane domains and cytoplasmic N-terminus and C-terminus (S1A Fig). Tagging GFP to the UNC-9 N-terminus affects its interaction with the gap junction regulator UNC-1, but UNC-9::GFP retains its endogenous localization and forms functional gap junctions [46]. Since UNC-9 has a rather short intracellular N-terminus (28 amino acids), we tested whether tagging GFP to its N-terminus will affect its function. We found that expression of GFP::UNC-9 under its own promoter (a 2.5kb fragment upstream of the start codon) rescued the uncoordinated phenotypes of *unc-9* mutants to a similar level as untagged UNC-9 (S1 Movie (*unc-9*), S2 Movie (*Punc-9*::*GFP*::*unc-9*(5ng/ul);*unc-9*), S3 Movie (*Punc-9*:: *unc-9*(5ng/ul);*unc-9*)), supporting the idea that GFP::UNC-9 fusion protein may function in the same manner as untagged UNC-9. Therefore, we examined UNC-9 localization using transgenes expressing GFP::UNC-9 in PLM neurons. We found that GFP::UNC-9 formed stereotypical patterns at two gap junction zones with 2–3 GFP puncta at zone 1 and one GFP puncta at zone 2 (Fig 1A), and the same expression pattern was also observed in UNC-9::GFP transgene (Fig 1A). Using an UNC-9 specific antibody [46], we confirmed that endogenous UNC-9 formed similar punctate structures as those observed transgenes (Fig 1B). We further confirmed those puncta localized at the region where PLM neurons meet their gap junction partners. As shown in S1B Fig, we expressed mCherry in PVC neurons (*Pglr-1*::*mCherry*) and consistently observed that one GFP::UNC-9 punctum localized to the region where PVC axons crossed PLM axons. *C*. *elegans* stomatin protein UNC-1 is co-localized with and functionally important for UNC-9 containing gap junctions in muscle cells [46]. Consistent with this observation, we found that UNC-1 formed similar punctate patterns in PLM neurons (Fig 1A). In addition to forming gap junctions, UNC-9 and UNC-7 could also function as hemichannels in *C*. *elegans* motor neurons [47]. The conserved cysteines (Cys) at extracellular loops are essential for formation of UNC-7/UNC-9 gap junctions but not hemichannels [47]. We found that mutating these cysteines (Cys) to alanines (Ala) blocked the formation of GFP::UNC-9 puncta, supporting the conclusion that those puncta were gap junctions but not hemichannels (S1C Fig). All together, we believe that the GFP puncta in GFP::UNC-9 transgenes represent the localization of PLM gap junctions.

Since UNC-1 is co-localized and functionally important for UNC-9 containing gap junctions [46], it might be involved in gap junction assembly. To verify this possibility, we tested the formation of GFP::UNC-9 puncta in *unc-1(lf)* background and found that loss of function of *unc-1* did not affect UNC-9 puncta, suggesting UNC-1 was not required for the formation of UNC-9 puncta in PLM neurons (Fig 1C). This observation is consistent with previous findings in muscle cells [46]. We also tested whether UNC-9 localization depended on *inx-7*, an innexin co-expressed in PLM neurons with *unc-9*. As shown in Fig 1C, we did not observe any defects of UNC-9 distribution in *inx-7* mutants, suggesting that the assembly of UNC-9 puncta did not rely on other gap junction proteins. Previous studies showed that Netrin and its receptor Frazzled could regulate the formation of gap junctions between Drosophila interneurons and motor neurons [48]. We tested whether the Netrin signaling pathway was also involved in formation of PLM gap junctions. We found that loss of function of *unc-6*, the only Netrin homolog in *C*. *elegans*, did not affect the formation of UNC-9 puncta. There are two Netrin receptors in *C*. *elegans*, UNC-5 and UNC-40/DCC. Loss of function of *unc-5* did not affect UNC-9 puncta, but about 3–5% *unc-40*/DCC*(e271)* mutant animals lost UNC-9 puncta (S1D Fig). Further studies will be necessary to confirm the function of UNC-40/DCC in gap junction regulation.

Gap junctions are required for the formation of chemical synapses in leeches [26]. The conserved Neurobeachin is involved in development of both chemical synapses and gap junctions in zebrafish [49]. It seems that the formation of gap junctions and chemical synapses could share some common mechanisms. To test this idea in *C*. *elegans*, we examined GFP::UNC-9 puncta in loss-of—function mutants of *rpm-1* and *syd-2*, two genes playing important roles in *C*. *elegans* chemical synapse formation [50–53] and found that neither of them was required for the organization of UNC-9 puncta (Fig 1D). Double mutants of *rpm-1* and *syd-2* suppress the formation of chemical synapses [54], but we did not observe any defects of UNC-9 puncta in double mutant animals (Fig 1D). These results are in support of different mechanisms regulating gap junction and chemical synapse formation in *C*. *elegans* neurons.

### UNC-44/ankyrin and UNC-33/CRMP regulate UNC-9 puncta

**Fig 2:**
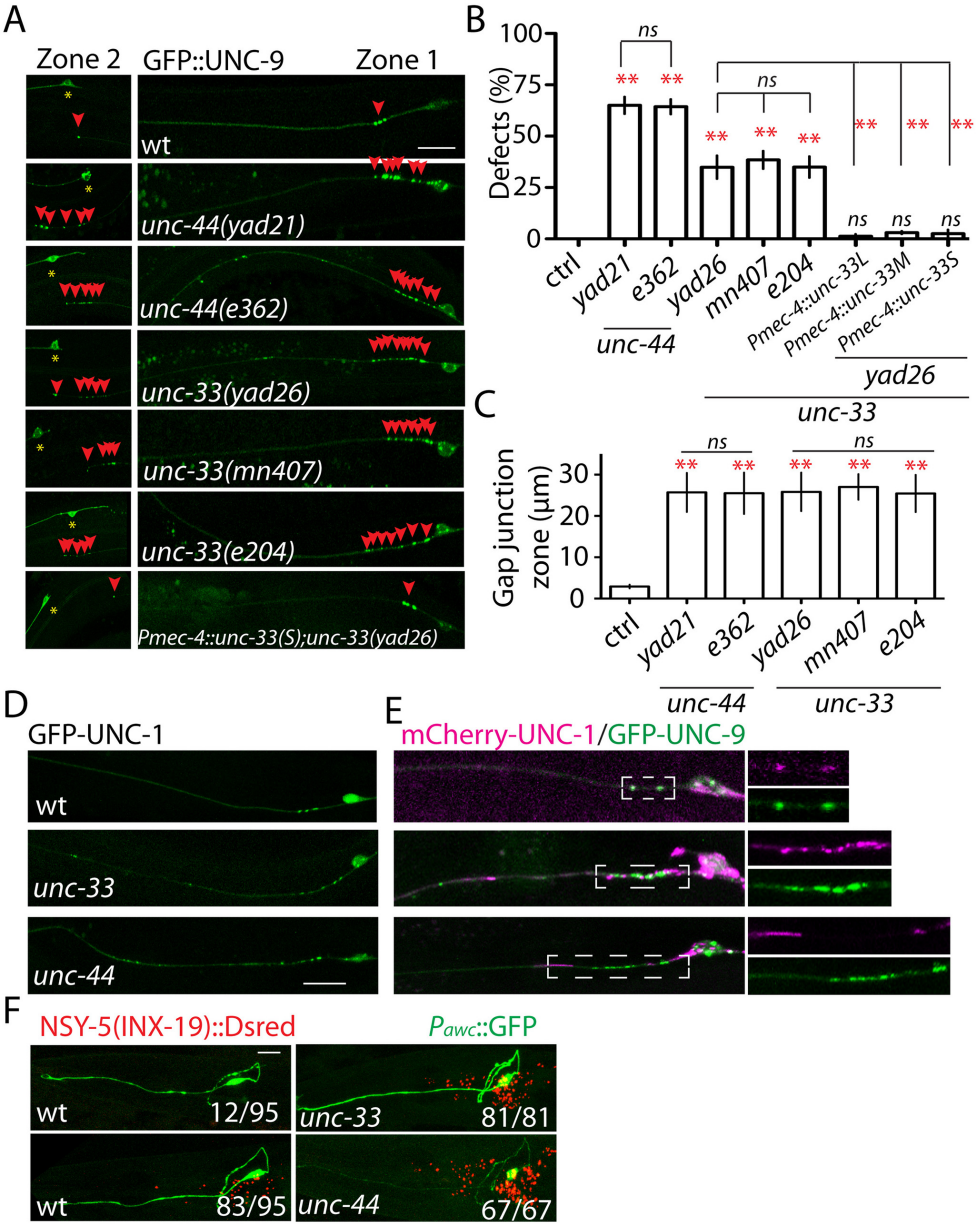
UNC-44 and UNC-33 regulate gap junctions. **(A)** Confocal images show GFP::UNC-9 puncta in *unc-33* and *unc-44* mutants, and *unc-33(lf)* rescue strain. **(B)** Quantification of the percentage of animals with more than four GFP::UNC-9 puncta at gap junction zone 1 in wild type and mutant strains. Each experiment was performed with N>200 animals at least three times. *Pmec-4* promoter was used to express *unc-33* cDNA in six mechanosensory neurons including PLM neurons. For transgenic animals the results shown here are generated from at least three independent lines. **(C)** Quantification of the length of gap junction zone 1 in wild type and mutants with enlarged gap junction zones. N>20 animals. Loss of function of *unc-33* or *unc-44* disrupts UNC-1 localization **(D)** and affects the co-localization of UNC-1 with UNC-9**(E)**. **(F)** Mutating *unc-33* and *unc-44* increase NSY-5(INX-19) puncta number in adult animals. The number displayed at each images shows the number of animals with showing phenotypes/ total animals. Data are shown as mean ± SD. In comparisons of two groups Student t-test was used. In comparison of three *unc-33* allele phenotypes, one-way ANOVA was used. ** p<0.01, ns: no significant difference. Scale bar: 10 μm.

To uncover the molecular mechanisms underlying gap junction regulation, we carried out an unbiased genetic screen using *yadIs12* (*Pmec-4*::*GFP*::*unc-9*) as a starting strain and isolated mutants with three types of phenotypes: 1, type one mutants lost UNC-9 puncta; 2, type two mutants had more UNC-9 puncta close to the original gap junctions; 3, type three mutants had more UNC-9 puncta along the axon (Fig 1E). In this study, we focused on two mutants with type two phenotypes. As shown in Fig 2A and 2B, about 65% of *yad21* and 35% of *yad26* animals had more UNC-9 puncta at both zone 1 and zone 2, and the length of gap junction zone 1 in those animals was enlarged from 2–3 μm to about 25 μm (Fig 2C). Neither *yad21* nor *yad26* changed the overall expression level of GFP::UNC-9 (S1F Fig). Both *yad21* and *yad26* were linked to *yadIs12* marker on Chromosome IV and had strong uncoordinated (*unc*) phenotypes. After testing some *unc* genes on chromosome IV, we found *yad21* failed to complement the loss-of-function allele of *unc-44(e362)*, and *yad26* failed to complement the loss-of-function allele of *unc-33(mn407)*. *unc-44(e362)* had the same UNC-9 punctate defects as that seen in *yad21*, and *unc-33(mn407)* and *unc-33(e204)* had identical phenotypes with *yad26* (Fig 2A and 2B). Sequencing results showed that *yad21* introduced a point mutation (P4813S) and a premature stop codon (Q6827*) in the neuronal specific long isoform of *unc-44*, and *yad26* had two point mutations (E168K and G328D) in *unc-33* (S2A and S2B Fig). These evidences supported the conclusion that *yad21* was a loss-of-function allele of *unc-44*, and *yad26* was a loss-of-function allele of *unc-33*.

*unc-44* and *unc-33* are homologs of ankyrins and CRMPs, respectively, and loss of function of them causes defects in axon development [36,39], raising a concern that the UNC-9 distribution defects could be an indirect result of misregulation of axon development. In majority of *yad21* (83% n = 220 animals) and *yad26* (94%, n = 175 animals) animals, PLM axons grew straight from cell bodies to the middle section of the body similar to wild type animals, but about 70% of *yad21(lf)* and *yad26(lf)* animals had either shorter or longer PLM axons (S3A Fig). However, we did not notice any correlation between the UNC-9 distribution defects and axon phenotypes. We quantified UNC-9 distribution defects in animals with normal axon length and found that the percentage of animals with UNC-9 distribution defects (*yad21*: 62% n = 71, *yad26*: 32.3% n = 62) was same as those seen in all animals. Loss of function of *unc-34*/Enabled/VASP affected PLM development. Loss-of-function mutation in *rpm-1* caused overextension of PLM axons, but we did not observe any defects of UNC-9 distribution in these mutants (Fig 1D and S1E Fig) [41]. These results showed that UNC-9 distribution defects in *unc-44(lf)* and *unc-33(lf)* were not results of axon growth defects.

The UNC-44/UNC-33 pathway regulates neuronal polarity without changing the overall morphology of neurons [41]. There are three isoforms of *unc-33* in *C*. *elegans*, named L (long), M (middle) and S (short) isoforms based on the length of cDNA, and only the long isoform could rescue *unc-33(lf)* polarity defects [41]. We found that expression of any of these three isoforms in PLM neurons rescued UNC-9 distribution phenotypes in *unc-33(lf)* animals, supporting the phenotypes we observed were not results of mis-regulation of neuronal polarity (Fig 2A and 2B). The successful rescue of *unc-33(lf)* phenotypes by expressing *unc*-33 cDNA in PLM neurons also showed that *unc-33* cell autonomously regulated UNC-9 puncta (Fig 2A and 2B).

Using the UNC-9 specific antibody, we observed similar mis-accumulation of endogenous UNC-9 in *unc-44* and *unc-33* mutants (S3B Fig). We also noticed that UNC-1 was mis-localized (Fig 2D) and lost its co-localization with UNC-9 in *unc-33(lf)* and *unc-44(lf)* mutants (Fig 2E). Since the interaction between UNC-1 and UNC-9 was important for gap junction function [46], these results suggested that *unc-44* and *unc-33* might regulate gap junction functions. During *C*. *elegans* development, sensory neurons form NSY-5(INX-19) contained transient gap junctions, and most of these gap junctions are eliminated in adults [27]. In day one adults, about 13% of control animals did not have any NSY-5 puncta, and 87% of control animals had 30–40 NSY-5 puncta (Fig 2F). In *unc-33(lf)* and *unc-44(lf)* mutants, we observed significantly more NSY-5 puncta (50–90 punca/animal) in all examined animals (Fig 2F). These results support that UNC-44 and UNC-33 are involved in regulating multiple gap junction channels in different neuronal types.

**Fig 3:**
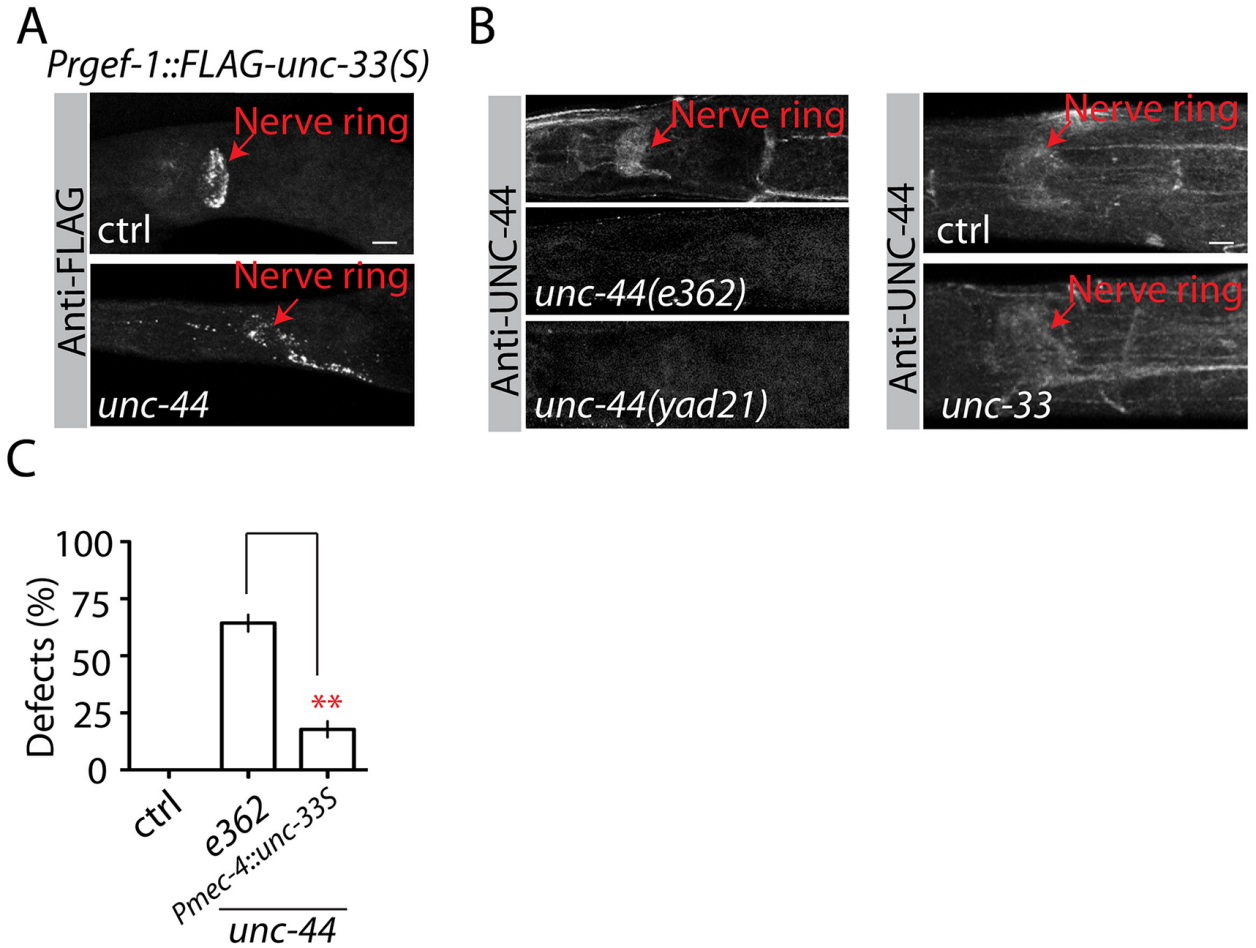
UNC-44 acts upstream of UNC-33. Representative images of immunostaining results for FLAG::UNC-33(S)**(A)** and UNC-44**(B)** in control and mutant animals. UNC-33(S) localization was determined by a transgene expressing FLAG tagged UNC-33(S) in all neurons (*Prgef-1*::*FALG*::*unc-33(S)*). **(C)** Overexpression of UNC-33(S) suppresses *unc-44(lf)* phenotypes. Each experiment was performed with N>200 animals at least three times. For transgenic animals the results shown here are generated from at least three independent lines. Data are shown as mean ± SD. Student’s t-test, ** p<0.01. Scale bar: 10 μm.

To determine whether *unc-44* and *unc-33* could regulate each other, we carried out immunostaining experiments. As shown in Fig 3A, UNC-33(S) accumulated at the nerve ring of control animals, and *unc-44* mutants induced more diffuse distribution of UNC-33(S). Using an antibody specifically recognizing the neuronal specific long isoform of UNC-44 [39], we found that UNC-44(L) was present in the nerve ring and neuronal processes, and *unc-33* loss-of-function mutants did not affect UNC-44 distribution (Fig 3B). These results were consistent with a previous report [41] and supported the hypothesis that UNC-44 acted upstream and regulated UNC-33 localization. We further confirmed this conclusion by testing the suppression ability of overexpressing *unc-33(S)* on *unc-44(lf)* phenotypes. As shown in Fig 3C, expressing *unc-33(S*) at high level suppressed *unc-44(lf)* phenotypes. In conclusion, by analyzing mutants with abnormal accumulation of UNC-9 in PLM neurons, we uncovered an important role of the UNC-44/UNC-33 pathway in the regulation of gap junctions.

### The UNC-44/UNC-33 pathway regulates UNC-9 dynamics and turnover

**Fig 4:**
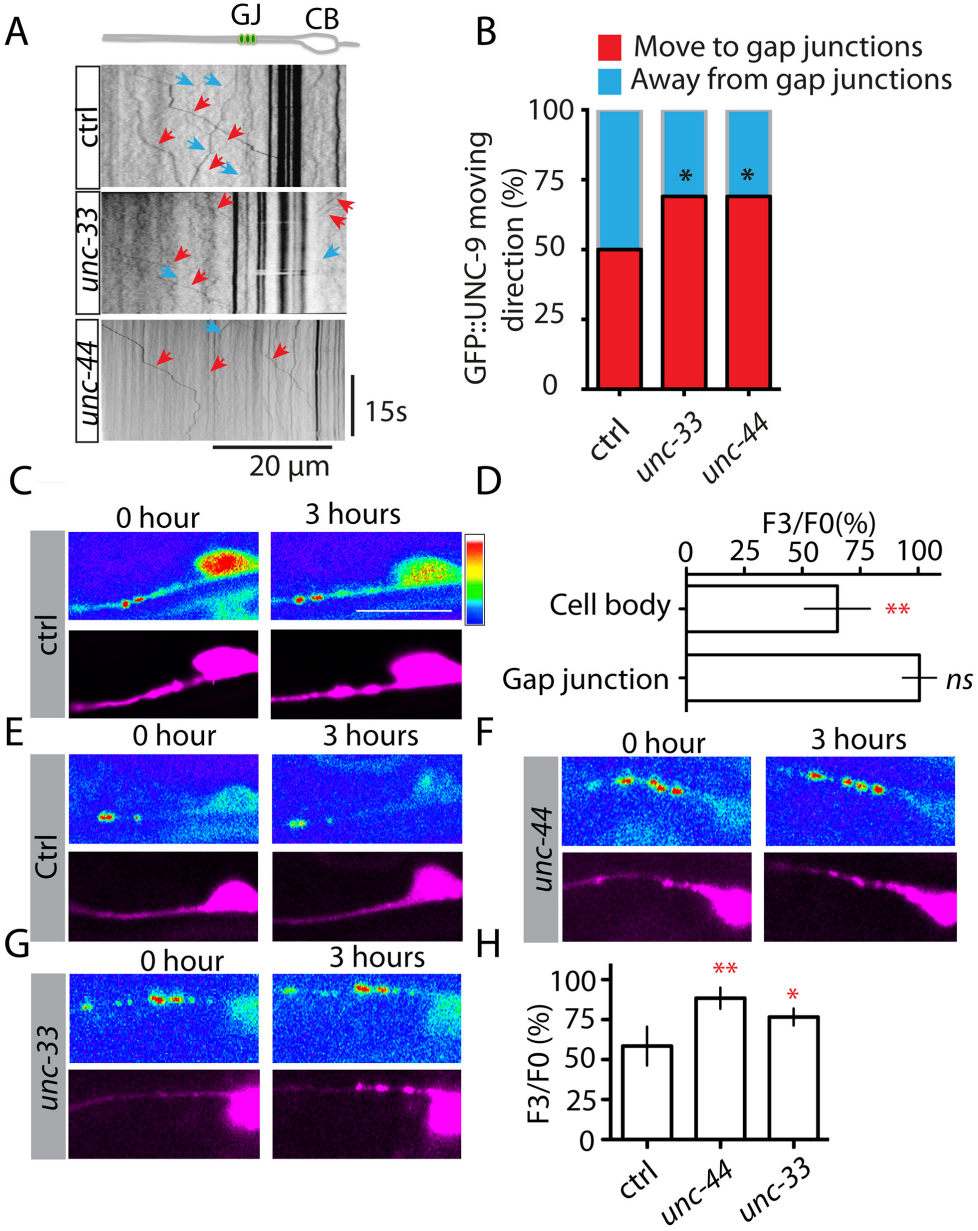
the UNC-44/UNC-33 pathway regulates gap junction dynamics and turnover. **(A)** Time kymographs generated from a 30s movie of GFP::UNC-9 dynamics in control, *unc-33(lf)* and *unc-44(lf)* animals. In the *unc-44* kymograph, the strong GFP::UNC-9 puncta were out of focus to visualize the GFP::UNC-9 movement. **(B)** Quantification of the percentage of particles moving to (red color) and moving away from gap junctions (blue color) in control (n = 42 animals), *unc-33*(n = 38) and *unc-44* (n = 38) based on the analysis of GFP::UNC-9 movement. Values that differ significantly from wild type (Fisher’s exact test) are denoted on the graphs (* p<0.05). Images **(C)** and quantification **(D)** show that photoactivation of PAGFP::UNC-9 at both cell bodies and axons induces 35% decrease of PAGFP::UNC-9 intensity at cell bodies after 3 hours, but does not change PAGFP::UNC-9 intensity at gap junction zone 1. The rainbow bar shows the color code for fluorescence intensity: from blue (low) to white (high). Images **(E-G)** and quantification **(H)** show that photoactivation of PAGFP::UNC-9 only at gap junction zone 1 induces 43% decrease of PAGFP::UNC-9 fluorescence intensity at gap junction zone 1 after 3 hours in control animals, and loss of function of *unc-44* and *unc-33* suppresses the decrease of PAGFP::UNC-9 intensity. In C, E, F and G the top panels show GFP signals from photoactivated PAGFP::UNC-9, and the bottom panels show mCherry signals from expression of *Pmec-4*::*mCherry* as internal controls. For quantification in D and H, F3/F0 was calculated based on: (PAGFP intensity/mCherry intensity 3 hours after photoactivation)/ (PAGFP intensity/mCherry intensity right after photoactivation)X100%, N>15 animals for each experiments. Data are shown as mean ± SD. Student’s t-test, ** p<0.01, * p<0.05, ns: no significant difference. Scale bar: 10 μm.

The striking phenotypes of *unc-44* and *unc-33* suggested that this pathway might regulate gap junction dynamics. To test this possibility, we analyzed GFP::UNC-9 movement at the gap junction zone one. In control animals, we observed bidirectional movement of UNC-9 both anterior and posterior to gap junctions, and each animal had almost equivalent numbers of UNC-9 particles moving toward and away from gap junctions (Fig 4A and 4B and S4 Movie), implicating the stable number of UNC-9 puncta at zone 1 was due to the balance of bidirectional movement. Loss-of-function mutation in *unc-33* or *unc-44* decreased the number of UNC-9 particles moving away from gap junctions and induced an imbalance of gap junction dynamics (Fig 4A and 4B, S5 and S6 Movies). These results suggested that *unc-44(lf)* and *unc-33(lf)* phenotypes might be due to suppression of gap junction turnover. To further examine this hypothesis, we used a transgene expressing photoactivatable GFP (PAGFP) tagged UNC-9 [55]. In this experiment, we first photoactivated PAGFP::UNC-9 in both cell bodies and axons, and we found that the fluorescence intensity at cell bodies decreased 35% at 3 hours after photoactivation, but the fluorescence intensity at gap junction zone 1 did not change in the same time period (Fig 4C and 4D). The decrease of fluorescence intensity at cell bodies was likely due to continuous transport of PAGFP::UNC-9 out of cell bodies. At gap junction zone 1, the stable fluorescence intensity indicated rapid turnover and replacement of PAGFP::UNC-9. In support of this conclusion, in animals that we locally photoactivated PAGFP::UNC-9 only at zone 1, we found that the fluorescence intensity decreased 43% after 3 hours in control (Fig 4E and 4H), and loss of function of *unc-44* and *unc-33* suppressed the decrease of fluorescence intensity (Fig 4F, 4G and 4H). We also tested whether *unc-44* and *unc-33* were involved in the assembly of UNC-9 puncta using the Fluorescence Recovery After Photobleaching (FRAP) assay. Briefly, we photobleached the GFP signal in zone 1 and measured the recovery of GFP signal after 3 hours. As shown in S4 Fig, loss of function of neither *unc-44* nor *unc-33* affected the recovery of GFP signal, supporting the UNC-44/UNC-33 pathway did not regulate the assembly of UNC-9 puncta. We believed that the UNC-44/UNC-33 pathway regulated gap junction turnover through suppression of transport of UNC-9 out of gap junctions.

### UNC-44 and UNC-33 regulate UNC-9 through VAB-8

**Fig 5:**
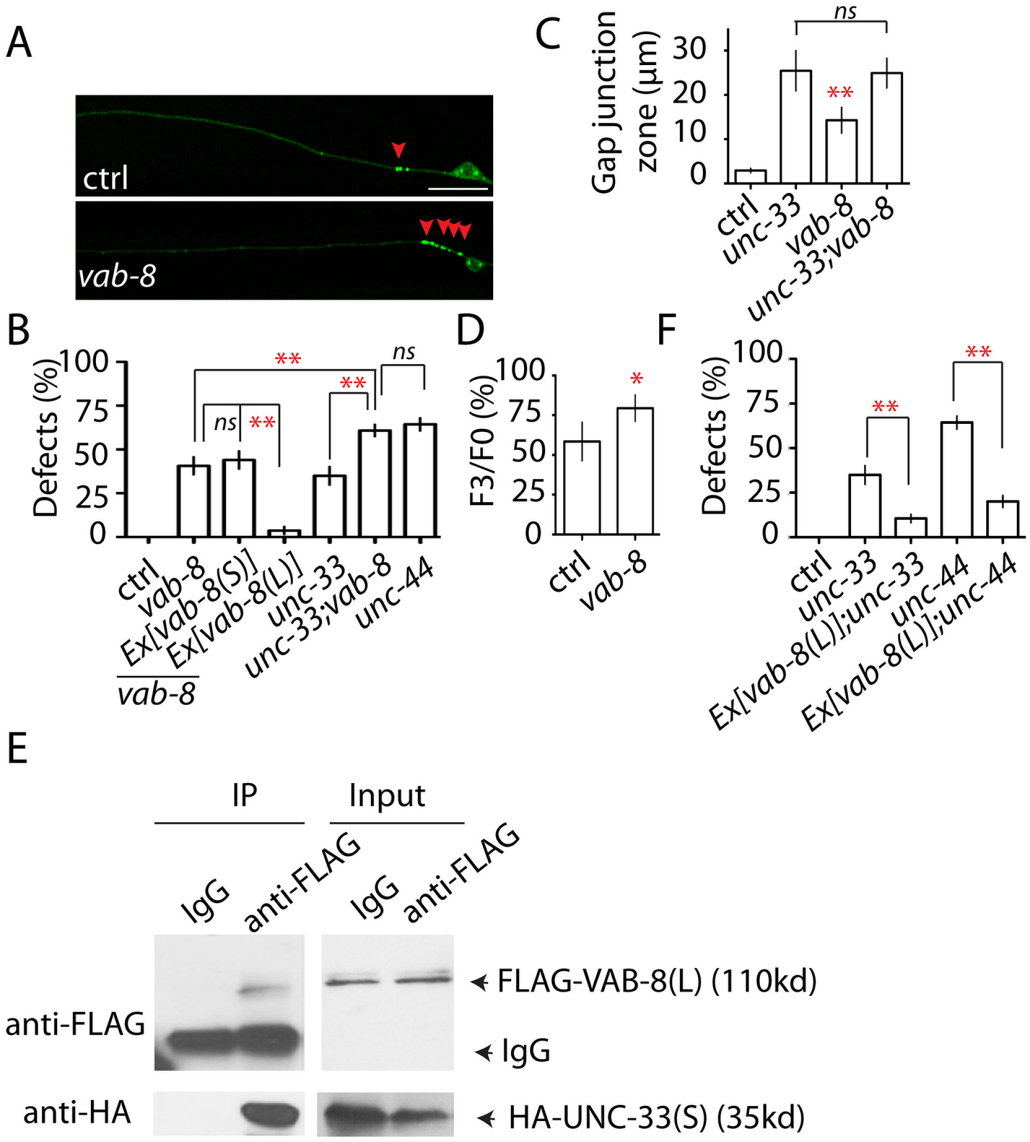
VAB-8 functions downstream of UNC-44 to regulate gap junction turnover. **(A)** Confocal images show the distribution of GFP::UNC-9 puncta in control and *vab-8(lf)* animals. **(B)** Quantification of the percentage of animals with more than four GFP::UNC-9 puncta at gap junction zone 1 in mutant and rescue strains. *Pmec-4* promoter was used to express *vab-8(L) and vab-8(S)* cDNA in PLM neurons. **(C)** Quantification of the length of gap junction zone 1 in wild type and mutants with enlarged gap junction zone. N>20 animals. **(D)** Quantification shows that after local photoactivation of PAGFP::UNC-9 at gap junction zone 1, the fluorescence intensity decreases about 43% at gap junction zone 1 in control animals, and loss of function of *vab-8* suppresses the decrease of PAGFP::UNC-9 intensity. N>15 animals. **(E)** VAB-8(L) binds to UNC-33(S) *in vivo*. **(F)** Overexpression of *vab-8(L)* suppresses *unc-33(lf)* and *unc-44(lf)* phenotypes. Each experiment was performed with N>200 animals at least three times. For transgenic animals the results shown here are generated from at least three independent lines. Data are shown as mean ± SD. Student’s t-test, ** p<0.01, * p<0.05, ns: no significant difference. Scale bar: 10 μm.

UNC-33 homolog CRMP-2 has been shown to bind to the kinesin light chain subunit kinesin-1 in transport of neurotrophin receptors into growth cones [56,57]. The effect of mutating *unc-44/unc-33* on UNC-9 dynamics suggested that they might regulate some motor proteins. We first tested three classic motor proteins, UNC-104, UNC-116 and DHC-1, that are known to be important for neuronal development. UNC-104 is the kinesin transporting synaptic vesicles from cell bodies to chemical synapses [58]. UNC-116 is a kinesin that is required for organization of presynaptic buttons and axonal mitochondria [59,60]. DHC-1 is the major dynein mediating retrograde transportation in *C*. *elegans* neurons [61]. However, we did not observe any UNC-9 defects caused by mutations in these three genes (S5 Fig). In testing the function of other motor proteins in UNC-9 regulation, we found that loss of function of a potential kinesin *vab-8* induced similar phenotypes as those seen in *unc-44(lf)* and *unc-33(lf)* (Fig 5A, 5B and 5C). Further genetic analysis showed that double mutants of *vab-8 unc-33* enhanced single mutant phenotypes to a degree similar to that seen in *unc-44* mutants, indicating that VAB-8 might work together with UNC-33 downstream of UNC-44 in regulating gap junction turnover (Fig 5B and 5C). Indeed, using the local photoactivation assay, we confirmed that loss of function of *vab-8* suppressed UNC-9 turnover (Fig 5D). *vab-8* has two isoforms, *vab-8(L)* and *vab-8(S)*, and the major difference between these two isoforms is that *vab-8(S)* lacks the kinesin domain present in *vab-8(L)* [62]. We found that only expression of *vab-8(L)* in PLM neurons was able to rescue *vab-8(lf)* phenotypes, suggesting the potential kinesin function of VAB-8 was essential for gap junction regulation (Fig 5B). Since UNC-33 homolog CRMP-2 has been shown to directly bind to kinesin, we examined whether UNC-33(S) could bind to VAB-8(L). As shown in Fig 5E, using a transgene pan-neurally expressing FLAG::VAB-8(L) and HA::UNC-33(S), we detected co-immunoprecipitation of UNC-33(S) and VAB-8(L), supporting that UNC-33(S) could bind to VAB-8(L) *in vivo*. This observation raised the possibility that UNC-33 might regulate the activity of VAB-8 in control of UNC-9 dynamics, such that, adding additional VAB-8L would restore UNC-9 distribution in *unc-33* and *unc-44* mutants. Indeed, we found that overexpression of VAB-8L in PLM neurons was able to suppress both *unc-33(lf)* and *unc-44(lf)* phenotypes (Fig 5F). These results showed that VAB-8L might function together with UNC-33 at the downstream of UNC-44 to regulate UNC-9 dynamics.

## Discussion

**Fig 6:**
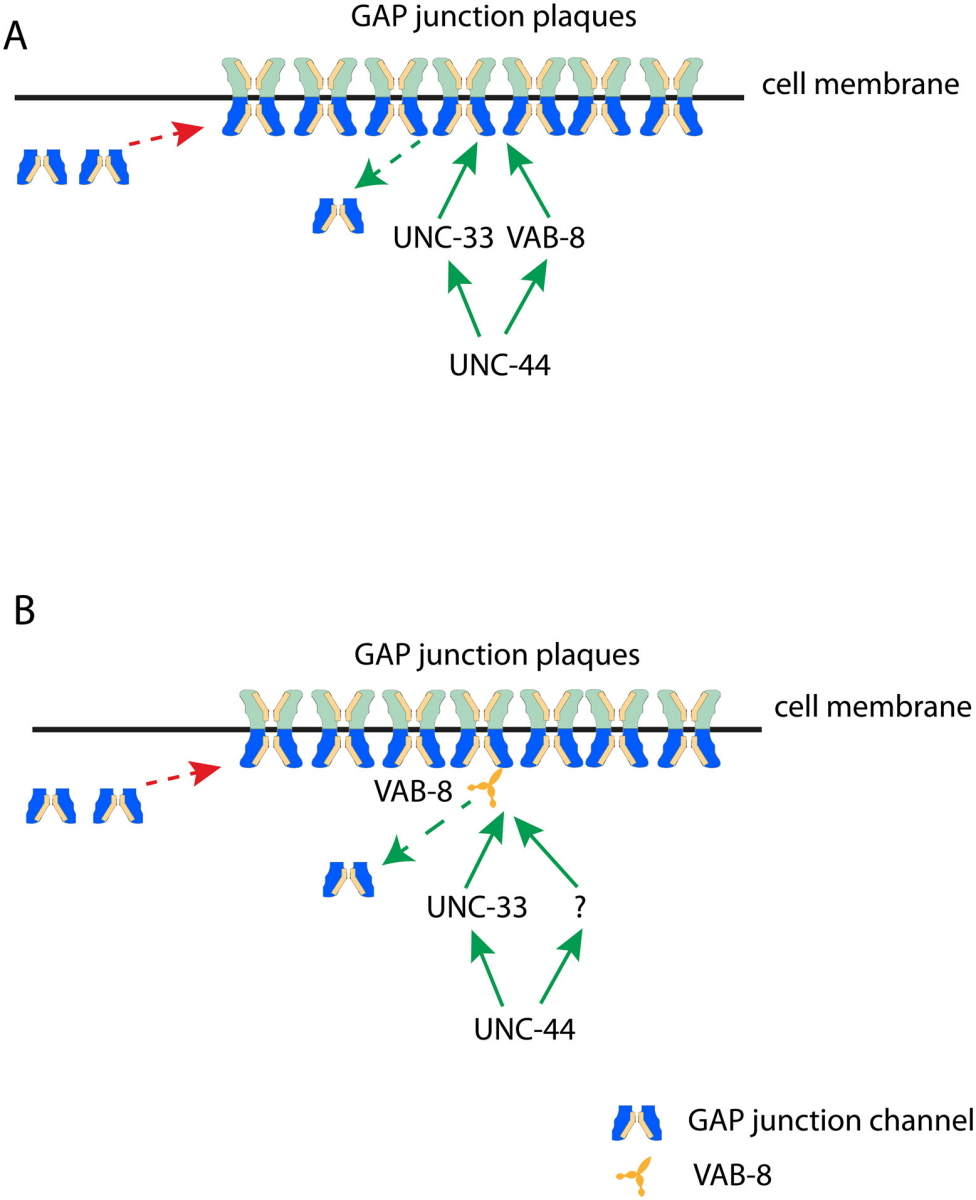
Models for the regulation of gap junctions the UNC-44/UNC-33/VAB-8 pathway. **(A)** VAB-8 and UNC-33 function in parallel at the downstream of UNC-44 in gap junction regulation. **(B)** In gap junction dynamics, UNC-33 works together with an unknown factor at the downstream of UNC-44 to regulate motor protein VAB-8 to control gap junction turnover.

Using *C*. *elegans* PLM neurons as a model, we investigated the molecular mechanisms underlying gap junction regulation. In adult animals, PLM neurons form gap junctions at two regions, at zone 1 with PVC, LUA and PVR neurons and at zone 2 with BDU neurons [42–44]. Using transgenes expressing GFP tagged UNC-9, we were able to observe punctate structures that represented the localization of gap junctions in PLM neurons. In a genetic screen targeting isolation of mutants affecting gap junctions, we uncovered the important roles of *C*. *elegans* ankyrin/*unc-44* and CRMP/*unc-33* in regulating gap junctions. By imaging GFP::UNC-9 dynamics and PAGFP::UNC-9 decay, we further demonstrated that the UNC-44/UNC-33 pathway regulated UNC-9 dynamics and turnover. In searching for motor proteins contributing to *unc-33/unc-44* phenotypes, we uncovered a role for a potential kinesin VAB-8 in UNC-9 turnover. VAB-8(L) directly bind to UNC-33(S), and overexpression of VAB-8(L) suppressed both *unc-33(lf)* and *unc-44(lf)* phenotypes, supporting the hypothesis that VAB-8 is the downstream target of the UNC-44 pathway in gap junction regulation. Double mutants of *vab-8* and *unc-33* had stronger phenotypes than each of single mutant and showed similar phenotypes as *unc-44(lf)*, suggesting that UNC-33 and VAB-8L function in parallel downstream of UNC-44. The suppression of *unc-33(lf)* phenotypes by VAB-8L overexpression was likely due to the enhanced contribution from VAB-8L (Fig 6A). The other possibility is that VAB-8 is regulated by both UNC-33 and an unknown factor that is in parallel with UNC-33 and downstream of UNC-44 (Fig 6B).

CRMPs bind to tubulin heterodimers and microtubules to promote the assembly of microtubules during neuronal polarity and to regulate Numb-mediated endocytosis at growth cones [33–35,63,64]. In all these cases, the phosphorylation of CRMPs by Rho kinase or GSK-3β is important for its regulation [33,34]. However, UNC-33(S), the minimal rescue fragment of UNC-33, does not have those phosphorylation sites, suggesting other mechanisms may be involved in regulating UNC-33/CRMP. Besides its roles in regulating microtubules, CRMP-2 has been shown to bind with the kinesin light chain subunit, kinesin-1, in transport of neurotrophin receptors into growth cones [56,57]. In cultured neurons, CRMP-2 forms a complex with Slp1 and Rad27B to directly link TrkB to kinesin-1, and to mediate the antrograde transportation of TrKB receptor upon stimulation of BDNF [56]. CRMP-2 can also mediate interactions between kinesin-1 and Sra-1/WAVE1 complex to control axon growth [57]. In our study, we found UNC-33/CRMP could bind to VAB-8L/Kinesin to regulate the dynamic and turnover of gap junction channels. These results suggest that the interactions between CRMPs and kinesins may play multiple roles in control of membrane proteins.

In our experiments, we found that the localization of UNC-33(S) was regulated by *unc-44*. Together with the suppression of *unc-44(lf)* phenotypes by overexpressing *unc-33(s)*, we concluded that *unc-44* acted upstream of *unc-33* in gap junction regulation. However, the localization of UNC-33(S) and UNC-44 appeared to be different, that UNC-33(S) was concentrated at the nerve ring, but UNC-44 had a broader distribution in neurons. One concern is that the transgene used to label UNC-33(S) (*Prgef-1*::*FLAG*:: *unc-33(S)*) may not reflect its *in vivo* localization. We compared our results of UNC-33(S) with the previous description of UNC-33(L) localization [41] and found that their localization were largely overlap, suggesting our transgene likely showed the *in vivo* localization of UNC-33(S). Since UNC-44 plays important roles in many aspects of neuronal development, the broad distribution of UNC-44 seems consistent with its multiple functions in neurons.

The UNC-44 homolog ankyrins have the ability to bind to channels and to integrate them to cytoskeleton, and the interactions between ankyrins and channels are dynamically regulated by intracellular signals [65]. It is possible that UNC-44 may play a role in holding UNC-9 at gap junctions. During gap junction turnover, the conformational change of UNC-44 could recruit UNC-33/CRMP and VAB-8L to UNC-9, and the activated UNC-33 could promote the transport of UNC-9 away from gap junctions through VAB-8L or an unknown factor.

## Materials and Methods

### *C*. *elegans* genetics

We maintained *C*. *elegans* strains on NGM plates at 20–22.5°C. All transgenes and strains are described in the S1 Table. We use *yadIs12* (Pmec-4::GFP::UNC-9) to visualize gap junctions in PLM neurons. Using *yadIs12* as a starting strain, we performed a clonal recessive screen following standard ethyl methane sulfonate (EMS) mutagenesis protocol. In 1000 mutagenized haploid genomes we examined, 12 mutants were isolated with three type phenotypes as mentioned in the manuscript.

### Cloning and constructs

All DNA expression constructs were made using Gateway cloning technology (Invitrogen). Sequences of the final clones were confirmed. The S1 Table lists the genotypes and DNA constructs for the transgenes. *unc-33* (L) cDNA was obtained from Dr. Cori. Bargmann, and *unc-33(M)* and *unc-33(S)* was amplified from *unc-33(L)* cDNA. *vab-8(L)* and *vab-8(S)* cDNA was amplified from yk clones from Dr. Yuji Kohara lab. Photoactivatable GFP (PAGFP) plasmid was obtained from Addgene. PAGFP coding sequence was inserted in an ASCI enzyme site before the start codon of UNC-9. All primer sequences are available upon request. Transgenic animals were generated following standard procedures. In general, plasmid DNAs of interest were used at 1–50 ng/ml with the co-injection marker *Pttx-3*::*rfp*/*Pttx-3*::*gfp* at 50 ng/μl.

### Fluorescence microscopy

Representative images and immunostaining results were acquired with a Zeiss LSM700 confocal microscope using a Plan-Apochroma 40x/1.4 objective. Worms were immobilized in 1% 1-phenoxy-2-propanol (TCI America, Potland, OR) in M9 buffer. For quantification of the percentage of animals with gap junction defects in PLM neurons, we used a Zeiss Axion Imager 2 microscope equipped with Chroma HQ filters. Each analyzed data performed takes at least three independent experiments and total 200–300 1-day old adults. For quantification of the length of gap junction zone, Images were acquired with LSM700 confocal microscope using a Plan-Apochroma 40x/1.4 objective, and the length of gap junction zone was measured using Zeiss Zen Black software.

### Photoactive experiments

The photoactive experiments were carried out with a Zeiss LSM700 confocal microscope using a Plan-Apochroma 40x/1.4 objective. 1-day young adult transgenes expressing mCherry and PAGFP::UNC-9 in PLM neurons were immobilized in 1% 1-phenoxy-2-propanol. We first use mCherry signal to localize PLM neurons and use 405 laser to globally or locally photoactivate PAGFP::UNC-9. After photoactive animals were recovered on the NGM plates for 3 hours before the second images were taken. The fluorescent intensity was analyzed using Image J software.

### Dynamic imaging

GFP::UNC-9 dynamic experiments were preformed using an Andor revolution microscopy with a 60 x /1.46 Plan-Apochromat objective controlled by MetaMorph software. All videos were acquired by an Andor EM-CCD camera (DU897). 1-day adult animals were immobilized in 5mM levamisole and on 5% agar pads for imaging. Videos for GFP::UNC-9 dynamic analysis were roughly 30–40s with 8 frames per second. Kymographs were generated using ImageJ, and the direction of GFP::UNC-9 movement was judged by the direction of the black lines in the kymograph pictures. In general, dynamic puncta were defined as their velocities >0.1 μm/s for last at least 5s. For puncta that change directions during experiments, we trace them for the overall direction.

### Fluorescence recovery after photobleaching (FRAP)

1-day adult animals were immobilized in 5mM levamisole and on 5% agar pads. FRAP was performed using Zeiss LSM700 confocal microscope using a Plan-Apochromat 40x/1.4 objective. A high-powered laser (at 100% energy, 488 nm) was used to photobleach the region of interest. Worms were recovered on NGM plates with food for 3 hours before taking the second image. Quantification of GFP::UNC-9 was carried out using Image J. Percentage Recovery = (I_3h_-I_bleach_)/(I_preblach_ -I_bleach_)x100. I_3h:_ the intensity at the region of interest (ROI) three hours after photobleaching; I_bleach_: the intensity at the ROI after photobleaching; I_prebleach:_ the intensity in the ROI before photobleaching. Background (the intensity in the non-bleached part of ROI) was subtracted, respectively.

### Immunostaining

All immunostaining experiments were carried out following the standard protocol using 1-day young adults. The Rabbit anti-UNC-9 antibody was a gift from Dr. Zhao-Wen Wang. The Rabbit anti-UNC-44(L) antibody was a gift from Dr. Anthony Otsuka. Mouse anti-FLAG M2 antibody (Cat# F1804), mouse anti- acetylated tubulin antibody (Cat# T7451) and rabbit anti-GFP (Cat# G1544) were purchased from Sigma. The dilutions for each antibodies are: anti-UNC-9(1:100), anti-UNC-44(1:100); anti-FLAG (1:300), anti-acetylated tubulin(1:300) and anti-GFP (1:150). Alexa Fluor 488 Donkey-anti-rabbit IgG (H+L) antibody (Cat# A-11008) and Alexa Fluor 594 Goat Anti-Mouse IgG (H+L) Antibody (Cat# A-11005) from Molecular Probes were used as secondary antibody in 1:500 dilution.

### Protein analysis

For the immunoprecipitation experiment, we generated an transgene (*yadEx421*) expressing FLAG-VAB-8(L) and HA-UNC-33(S) under the control of Pan-neuronal promoter *Prgef-1*. Proteins from mixed stages animals were first extracted using RIPA buffer by frozen-throw about 50 times in ethanol with dry ice, and protein lysis was incubated with mouse anti-FLAG M2 antibody (Cat# F1804) in room temperature for 5 hours and then precipitated using Protein A/G PLUS-Agarose (Santa Cruz Bio. esc-2003). Heated Protein samples were separated using SDS-PAGE Gradient Gels (4–20%), and then transferred to nitrocellulose. Blots were probed with mouse anti-Flag antibodies (sigma, F1804) and rabbit anti-HA(Sigma H6908), and then visualized with Amerisham HRP-conjugated anti-rabbit secondary antibodied at 1:5000 using the SuperSignal West Femto kit (Pierce, Rockford, 1L).

### Statistical analysis

We analyzed our data using one-tailed Student’s t test, one way ANOVA or Fisher exact test in Graphpad Prism (GraphPad Software, La Jolla, CA).

## Supporting Information

S1 Table Strains used in this study. (DOCX)

S1 Fig UNC-9 localization represents gap junctions in PLM neurons. (**A**) A cartoon shows the predicted structure of UNC-9, that UNC-9 has four transmembrane domains, two extracellular and one intracellular loop, and intracellular N- and C- termini. (**B**) GFP::UNC-9 puncta localized to PLM-PVC junctions. PVC axons was visualized by expressing *Pglr-1*::mCherry. An enlarged view of PLM-PVC gap junctions is displayed at the right side. (**C**) Mutating four conserved Cys required for UNC-9 gap junction functions alters UNC-9 localization. (**D**) Loss of function of Netrin (*unc-6*) or its receptor *unc-5* did not affect UNC-9 plaques, but loss of function of DCC(*unc-40*) suppressed the formation of UNC-9 plaques in about 3–5% animals. (**E**) Loss of function of *unc-34* did not change UNC-9 puncta. (**F**) Loss of function of *unc-33* or *unc-44* did not change GFP-UNC-9 expression level. GFP signal from expression of co-injection marker *Pttx-3*::GFP was used as the loading control. The number displayed at each images shows the number of animals with wild type GFP::UNC-9 plaques/ total animals. Scale bar: 10 μm.
(PDF)

S2 Fig Mutations identified in *yad21* (A) and *yad26* (B). (PDF)

S3 Fig Loss of function of *unc-44* and *unc-33* induced similar UNC-9 phenotypes as those seen in transgenes. **(A)** Representative images shows axons morphology of PLM neurons in control, *unc-44(yad21)* and *unc-33(yad26)*. For *unc-44* and *unc-33*, the examples of “wild type”, shorter and longer axons were displayed from top to bottom. The “length” of axons was defined by the relative positions between PLM axon terminals (red arrowheads) and ALM cell bodies (yellow stars). The number displayed at each images shows the number of animals with according phenotypes/ total animals. **(B)** Representative images of immunostaining results for UNC-9(green) and acetylated tubulin (Pink) in *unc-44(lf)* and *unc-33(lf)* animals. CB: cell bodies. Scale bar: 10 μm.
(PDF)

S4 Fig *unc-33* and *unc-44* do not affect the assembly of UNC-9 puncta. Image**(A)** and quantification**(D)** show that the fluorescence intensity at zone 1 recover 20% 3 hours after photobleaching. The thermal bar shows the color code for fluorescence intensity from purple (low) to red (high). Images**(B, C)** and quantification**(D)** show that loss of function of *unc-33* or *unc-44* doesn’t affect the recovery after photobleaching. N>10 animals for each experiments. Data are shown as mean ± SD. ns: no significant difference. Scale bar: 10 μm.
(PDF)

S5 Fig The organization of UNC-9 plaques does not rely on classic neuronal motor proteins. Loss of function of neuronal kinesin *unc-104* and *unc-116*, and dynein heavy chain *dhc-1* does not affect UNC-9 plaques. The number displayed at each images shows the number of animals with wild type GFP::UNC-9 plaques/ total animals. Scale bar: 10 μm.
(PDF)

S1 Movie Locomotion of *unc-9(e101)* animals. (MP4)

S2 Movie Locomotion of *Punc-9*::GFP::UNC-9;*unc-9(e101)* animals (5ng/ul). (MP4)

S3 Movie Locomotion of *Punc-9*:: UNC-9;*unc-9(e101)* animals (5ng/ul). (MP4)

S4 Movie GFP::UNC-9 dynamics in control animals. (AVI)

S5 Movie GFP::UNC-9 dynamics in *unc-33(lf)* animals. (AVI)

S6 Movie GFP::UNC-9 dynamics in *unc-44(lf)* animals (The strong GFP::UNC-9 puncta were out of focus to visualize the GFP::UNC-9 movement). (AVI)
