# Supplementary material for: Regulation of Gap Junction Dynamics by UNC-44/ankyrin and UNC-33/CRMP through VAB-8 in C. elegans Neurons
Source: PLoS Genet. 2016 Mar 25;12(3):e1005948. doi: 10.1371/journal.pgen.1005948 (PMC4807823; doi:10.1371/journal.pgen.1005948)
Supplement: S1 Table — (DOCX) [file pgen.1005948.s001.docx]

S1 Table

Strains used in this study

| Strain | Genotype | Plasmid | Related Figures |
| --- | --- | --- | --- |
| NYL413 | *Pmec-4::GFP-unc-9(cDNA)(yadIs12) IV* | PNYL185 | Fig.1a, and other, Control for all GFP::UNC-9 analysis |
| NYL737 | *Pmec-4::unc-9-GFP(yadEx334)* | PNYL187 | Fig.1A |
| NYL793 | *Pmec-4::GFP-unc-1(yadEx355)* | PNYL190 | Fig.1A,2D |
| NYL401 | *Pmec-4::GFP-unc-9(cDNA)(yadIs12) IV ; unc-1(e580null) X* |  | Fig.1C |
| NYL438 | *inx-7(ok2319) Pmec-4::GFP-unc-9(cDNA)(yadIs12) IV* |  | Fig.1C |
| NYL402 | *Pmec-4::GFP-unc-9(cDNA)(yadIs12) IV ; rpm-1(ju44ts) V* |  | Fig.1D |
| NYL408 | *Pmec-4::GFP-unc-9(cDNA)(yadIs12) IV ; syd-2(ju37) X* |  | Fig.1D |
| NYL415 | *Pmec-4::GFP-unc-9(cDNA)(yadIs12) IV; rpm-1(ju44ts) V; syd-2(ju37) X* |  | Fig.1D |
| NYL419 | *yad21(unc-44) Pmec-4::GFP-unc-9(cDNA)(yadIs12) IV* |  | Fig. 1E,2A,2B,2C,4A,4B,S4C |
| NYL444 | *unc-44(e362) Pmec-4::GFP-unc-9(cDNA)(yadIs12) IV* |  | Fig. 2A,2B,2C |
| NYL518 | *unc-33(yad26) Pmec-4::GFP-unc-9(cDNA)(yadIs12) IV* |  | Fig. 2A,2B,2C,4A,4B,S4B |
| NYL577 | *unc-33(mn407) Pmec-4::GFP-unc-9(cDNA)(yadIs12) IV* |  | Fig. 2A,2B,2C |
| NYL442 | *unc-33(e204) Pmec-4::GFP-unc-9(cDNA)(yadIs12) IV* |  | Fig. 2A,2B,2C |
| NYL496 | *unc-33(yad26) Pmec-4::GFP-unc-9(cDNA)(yadIs12) IV; Pmec-4::unc-33(cDNA S)(yadEx202)* | PNYL191 | Fig. 2A,2B |
| NYL492 | *unc-33(yad26) Pmec-4::GFP-unc-9(cDNA)(yadIs12) IV; Pmec-4::unc-33(cDNA L)* | PNYL193 | Fig. 2B |
| NYL494 | *unc-33(yad26) Pmec-4::GFP-unc-9(cDNA)(yadIs12) IV; Pmec-4::unc-33(cDNA M)* | PNYL192 | Fig. 2B |
| NYL915 | *unc-33(e204) IV; Pmec-4::GFP-unc-1(yadEx355)* |  | Fig. 2D |
| NYL913 | *unc-44(e362) IV; Pmec-4::GFP-unc-1(yadEx355)* |  | Fig. 2D |
| NYL1109 | *Pmec-4::GFP-unc-9(CDNA)(yadIs12) IV; Pmec-4::mcherry-unc-1(yadEx525)* | PNYL389 | Fig. 2E |
| NYL1118 | *unc-33(e204) Pmec-4::GFP-unc-9(CDNA)(yadIs12) IV; Pmec-4::mcherry-unc-1(yadEx525)* |  | Fig. 2E |
| NYL1119 | *Unc-44(e362) Pmec-4::GFP-unc-9(CDNA) (yadIs12) IV; Pmec-4::mcherry-unc-1 (yadEx525)* |  | Fig. 2E |
| CX7819 | *Pnsy-5::nsy-5(cDNA)::DsRed::nsy-5 5’UTR + Pelt-2::GFP (kyEx1222)* |  | Fig. 2F |
| NYL920 | *unc-33(e204) IV; Pnsy-5::nsy-5(cDNA)::DsRed::nsy-5 5’UTR + Pelt-2::GFP (kyEx1222)* |  | Fig. 2F |
| NYL919 | *unc-44(e362) IV; Pnsy-5::nsy-5(cDNA)::DsRed::nsy-5 5’UTR + Pelt-2::GFP (kyEx1222)* |  | Fig. 2F |
| NYL571 | *Prgef-1::FLAG-unc-33(s)(yadEx260)* |  | Fig. 3A |
| NYL610 | *unc-44(e362) IV; Prgef-1::FLAG-unc-33(s)(yadEx260)* |  | Fig. 3A |
| NYL937 | *Pmec-4:unc-33(S); unc-44(e362) IV (yadEx435)* |  | Fig. 3C |
| NYL601 | *Pmec-4::PAgfp-unc-9 + Pmec-4::mCherry(yadEx268)* | PNYL196 | Fig. 4C,4D,4E,4H |
| NYL616 | *unc-44(e362) IV; Pmec-4::PAgfp-unc-9 + Pmec-4::mCherry(yadEx268)* |  | Fig. 4F,4H |
| NYL615 | *unc-33(e204) IV; Pmec-4::PAgfp-unc-9 + Pmec-4::mCherry(yadEx268)* |  | Fig. 4G,4H |
| NYL524 | *Pmec-4::GFP-unc-9(cDNA)(yadIs12) IV; vab-8(e1017) V* |  | Fig. 5A,5B,5C |
| NYL599 | *Pmec-4::GFP-unc-9(cDNA)(yadIs12) IV; vab-8(e1017) V; Pmec-4::vab-8(S)(yadEx266)* | PNYL198 | Fig. 5B |
| NYL664 | *Pmec-4::GFP-unc-9(cDNA)(yadIs12) IV; vab-8(e1017) V; Pmec-4::vab-8-L(yadEx301)* | PNYL199 | Fig. 5B |
| NYL597 | *unc-33(e204) Pmec-4::GFP-unc-9(cDNA)(yadIs12) IV; vab-8(e1017) V* |  | Fig. 5B, 5C |
| NYL609 | *vab-8(e1017) V; Pmec-4::PAgfp-unc-9 + Pmec-4::mCherry(yadEx268)* |  | Fig. 5D |
| NYL901 | *Prgef-1::FLAG-vab-8(L) + Prgef-1::HA-unc-33(S) (yadEx421)* |  | Fig. 5E |
| NYL907 | *unc-33(e204) IV; Pmec-4::vab-8(L)(yadEx427)* |  | Fig. 5F |
| NYL935 | *unc-44(e362) IV; Pmec-4::vab-8(L)(yadEx433)* |  | Fig. 5F |
| NYL728 | *Pmec-4::GFP-unc-9(cDNA)(yadIs12) IV; Pglr-1::mCherry(yadEx329)* | PNYL226 | Fig. S1B |
| NYL721 | *Pmec-4::GFP-unc-9 (C/A)(yadEx326)* | PNYL202 | Fig. S1C |
| NYL475 | *unc-5(e53) Pmec-4::GFP-unc-9(cDNA)(yadIs12) IV* |  | Fig. S1D |
| NYL474 | *Pmec-4::GFP-unc-9(cDNA)(yadIs12) IV; unc-6(ev400) X* |  | Fig. S1D |
| NYL476 | *unc-40(e271) I; Pmec-4::GFP-unc-9(cDNA)(yadIs12) IV* |  | Fig. S1D |
| NYL443 | *Pmec-4::GFP-unc-9(cDNA)(yadIs12) IV; unc-34(e315) V* |  | Fig. S1E |
| CZ10175 | *Pmec-4::GFP(zdIs5) I* |  | Fig. S3A |
| NYL1030 | *Pmec4-GFP(zdIs5) I; unc-44(yad21) IV* |  | Fig. S3A |
| NYL1031 | *Pmec4-GFP(zdIs5) I; unc-33(yad26) IV* |  | Fig. S3A |
| NYL400 | *unc-104(rh43) II; Pmec-4::GFP-unc-9(cDNA)(yadIs12) IV* |  | Fig. S5 |
| NYL403 | *unc-116(e2310) III; Pmec-4::GFP-unc-9(cDNA)(yadIs12) IV* |  | Fig. S5 |
| NYL648 | *dhc-1(js319) I; Pmec-4::GFP-unc-9(cDNA)(yadIs12) IV* |  | Fig. S5 |
| NYL1128 | *unc-9(e101) X; Punc-9::GFP-unc-9(cDNA)(yadEx557)* | PNYL384 | MovieS2 |
| NYL1128 | *unc-9(e101) X; Punc-9:: unc-9(cDNA)(yadEx555)* | PNYL385 | MovieS3 |
