# Supplementary figures and images for: Regulation of Gap Junction Dynamics by UNC-44/ankyrin and UNC-33/CRMP through VAB-8 in C. elegans Neurons
Source: PLoS Genet. 2016 Mar 25;12(3):e1005948. doi: 10.1371/journal.pgen.1005948 (PMC4807823; doi:10.1371/journal.pgen.1005948)

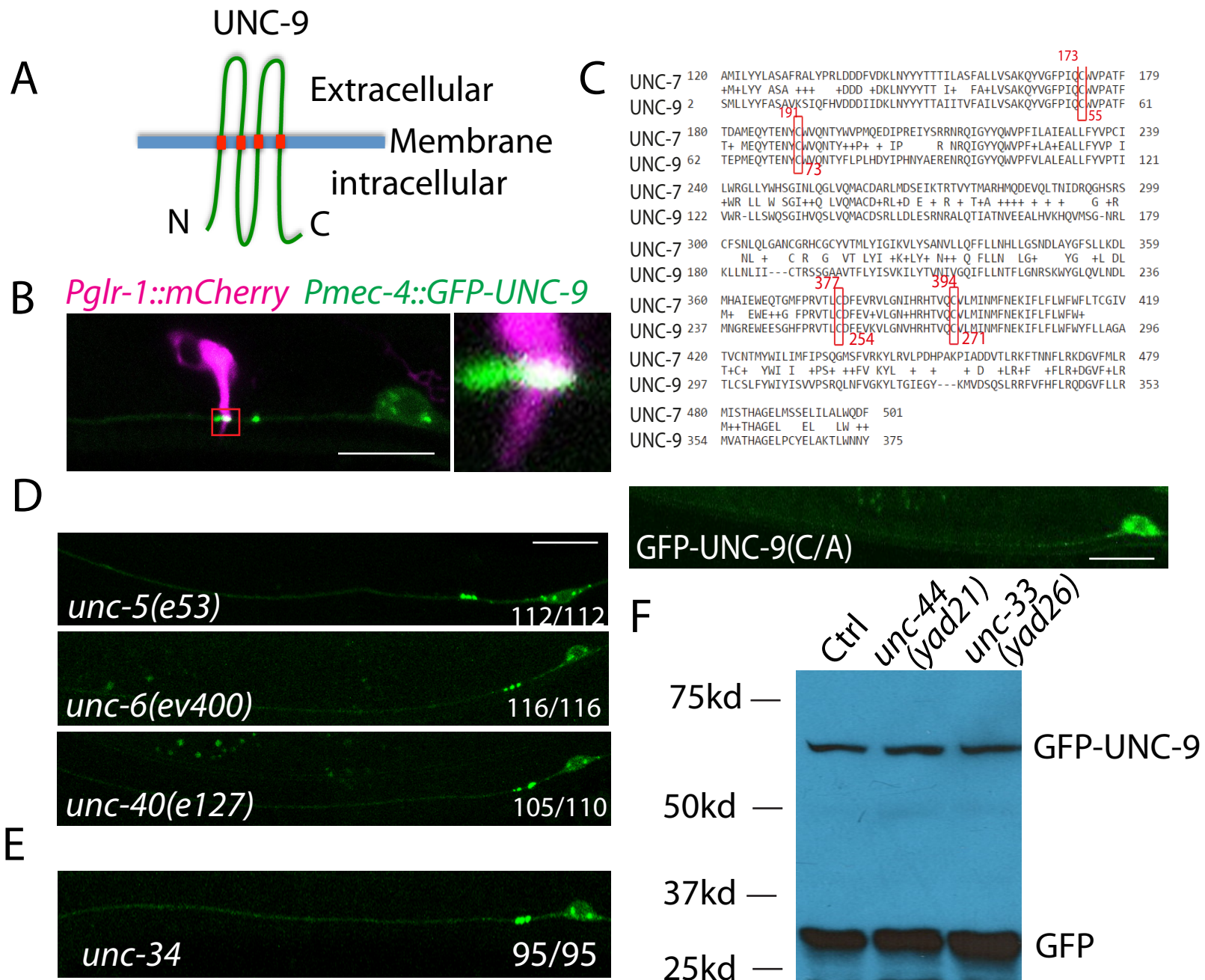

Supplement: S1 Fig — (A) A cartoon shows the predicted structure of UNC-9, that UNC-9 has four transmembrane domains, two extracellular and one intracellular loop, and intracellular N- and C- termini. (B) GFP::UNC-9 puncta localized to PLM-PVC junctions. PVC axons was visualized by expressing Pglr-1::mCherry. An enlarged view of PLM-PVC gap junctions is displayed at the right side. (C) Mutating four conserved Cys required for UNC-9 gap junction functions alters UNC-9 localization. (D) Loss of function of Netrin (unc-6) or its receptor unc-5 did not affect UNC-9 plaques, but loss of function of DCC(unc-40) suppressed the formation of UNC-9 plaques in about 3–5% animals. (E) Loss of function of unc-34 did not change UNC-9 puncta. (F) Loss of function of unc-33 or unc-44 did not change GFP-UNC-9 expression level. GFP signal from expression of co-injection marker Pttx-3::GFP was used as the loading control. The number displayed at each images shows the number of animals with wild type GFP::UNC-9 plaques/ total animals. Scale bar: 10 μm. (PDF) [file pgen.1005948.s002.pdf]

A

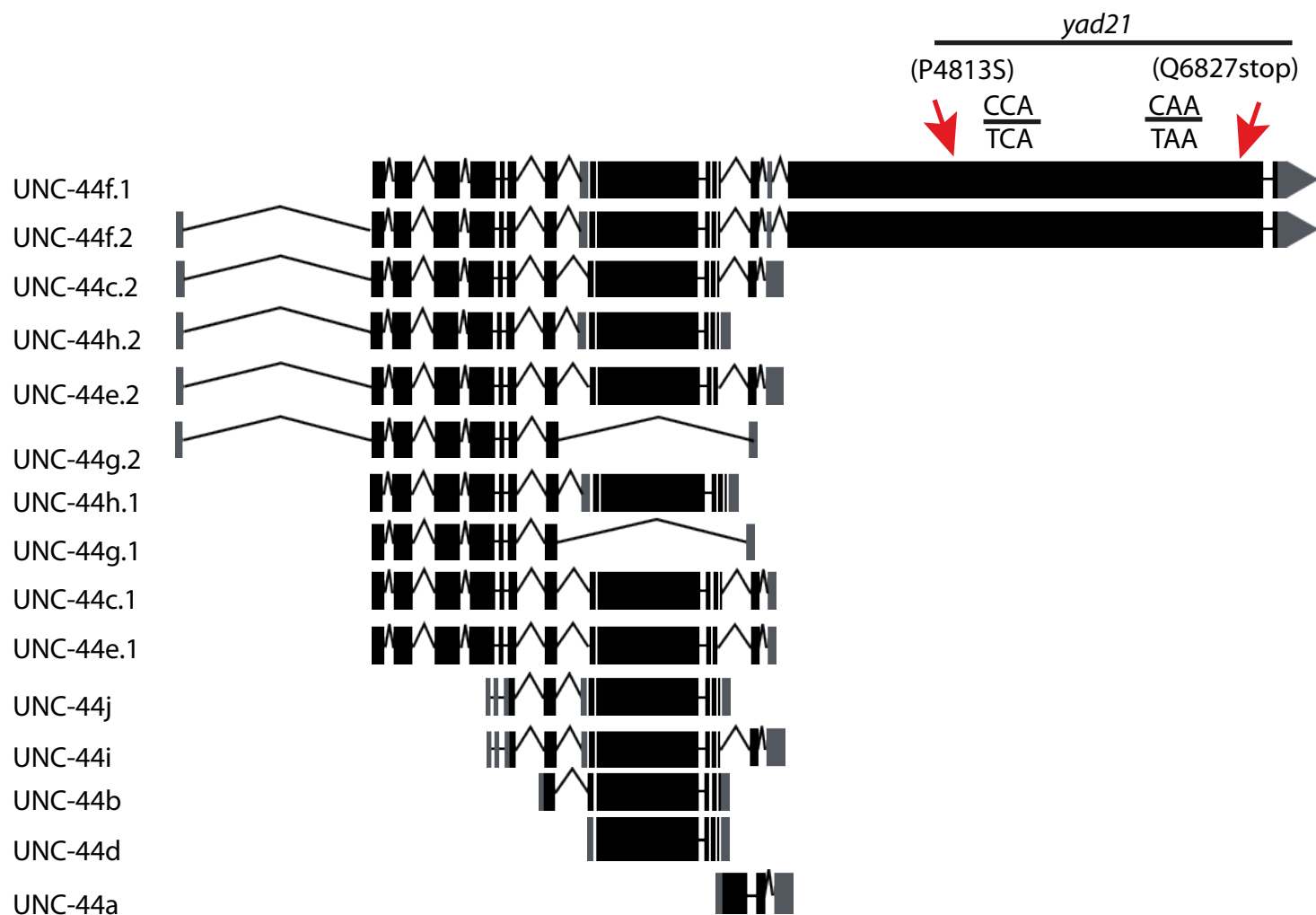

1 kb

B

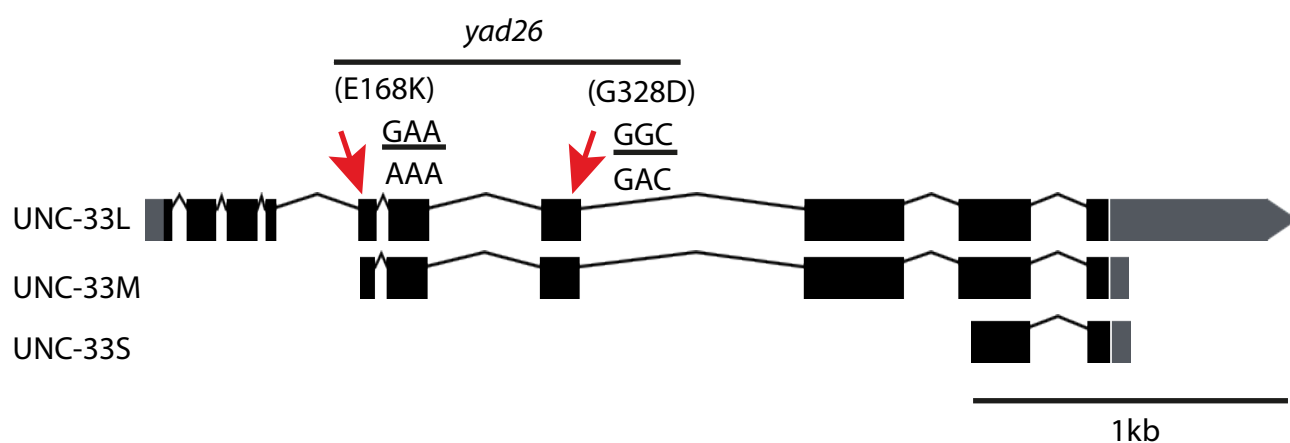

1kb

Supplement: S2 Fig — (PDF) [file pgen.1005948.s003.pdf]

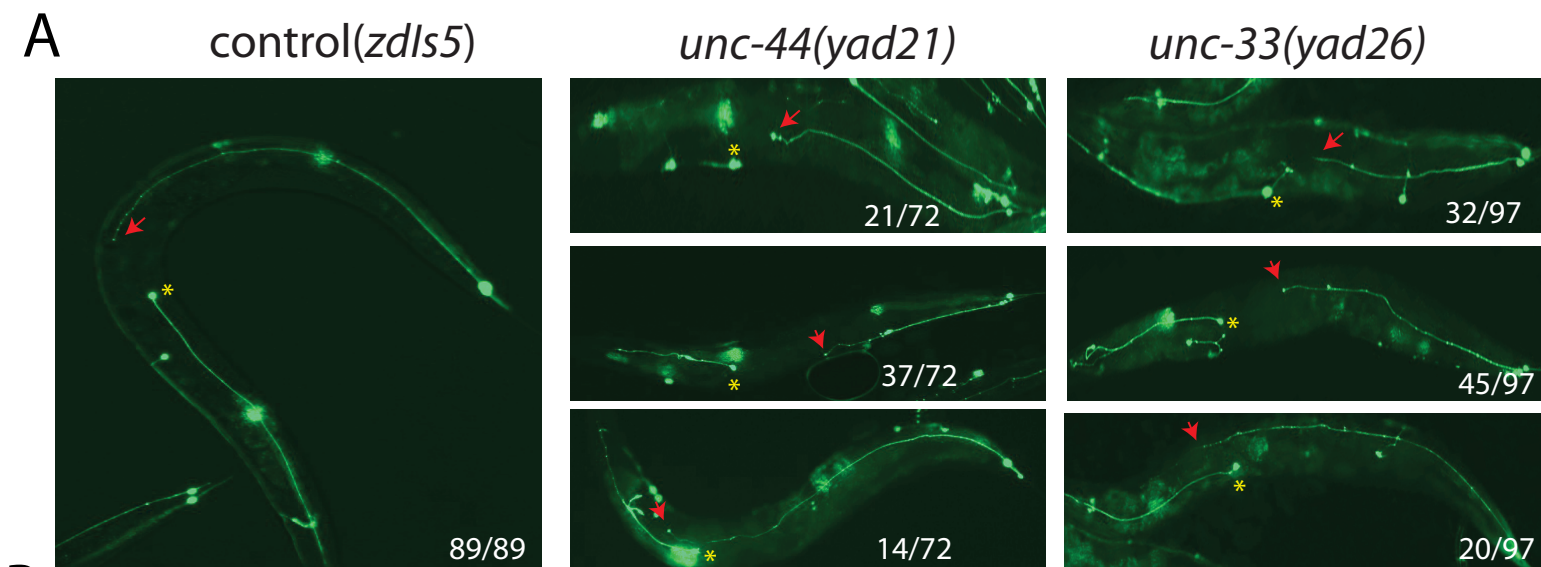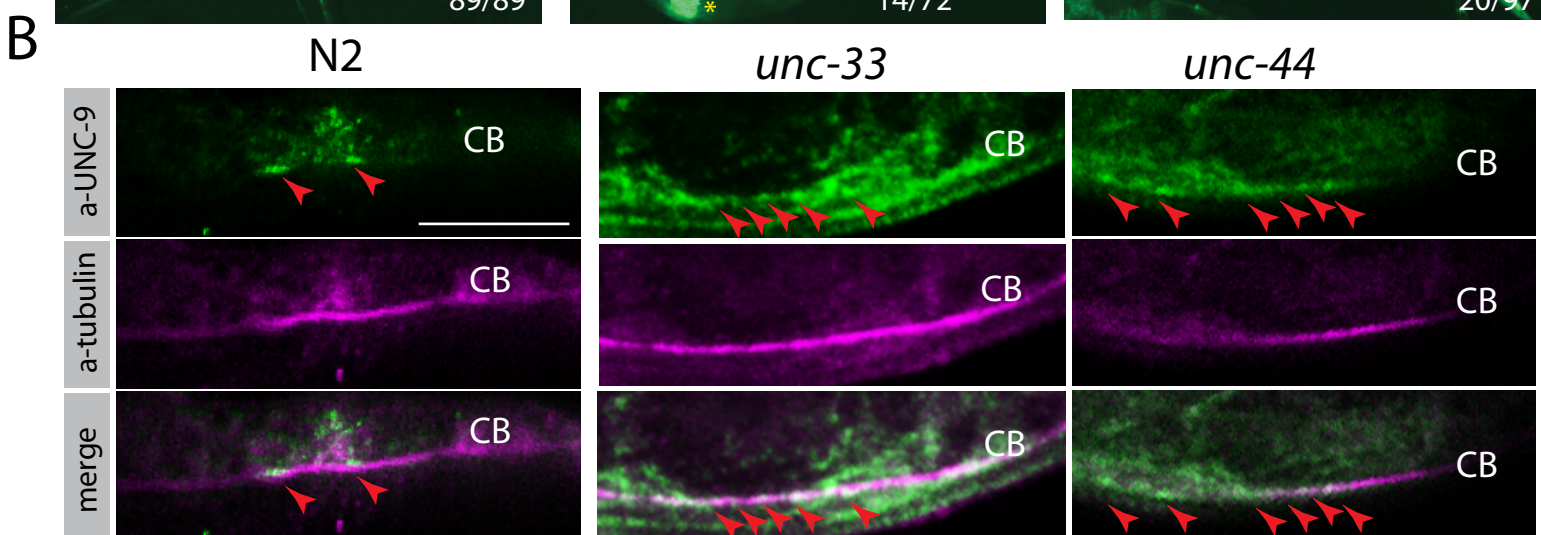

Supplement: S3 Fig — (A) Representative images shows axons morphology of PLM neurons in control, unc-44(yad21) and unc-33(yad26). For unc-44 and unc-33, the examples of “wild type”, shorter and longer axons were displayed from top to bottom. The “length” of axons was defined by the relative positions between PLM axon terminals (red arrowheads) and ALM cell bodies (yellow stars). The number displayed at each images shows the number of animals with according phenotypes/ total animals. (B) Representative images of immunostaining results for UNC-9(green) and acetylated tubulin (Pink) in unc-44(lf) and unc-33(lf) animals. CB: cell bodies. Scale bar: 10 μm. (PDF) [file pgen.1005948.s004.pdf]

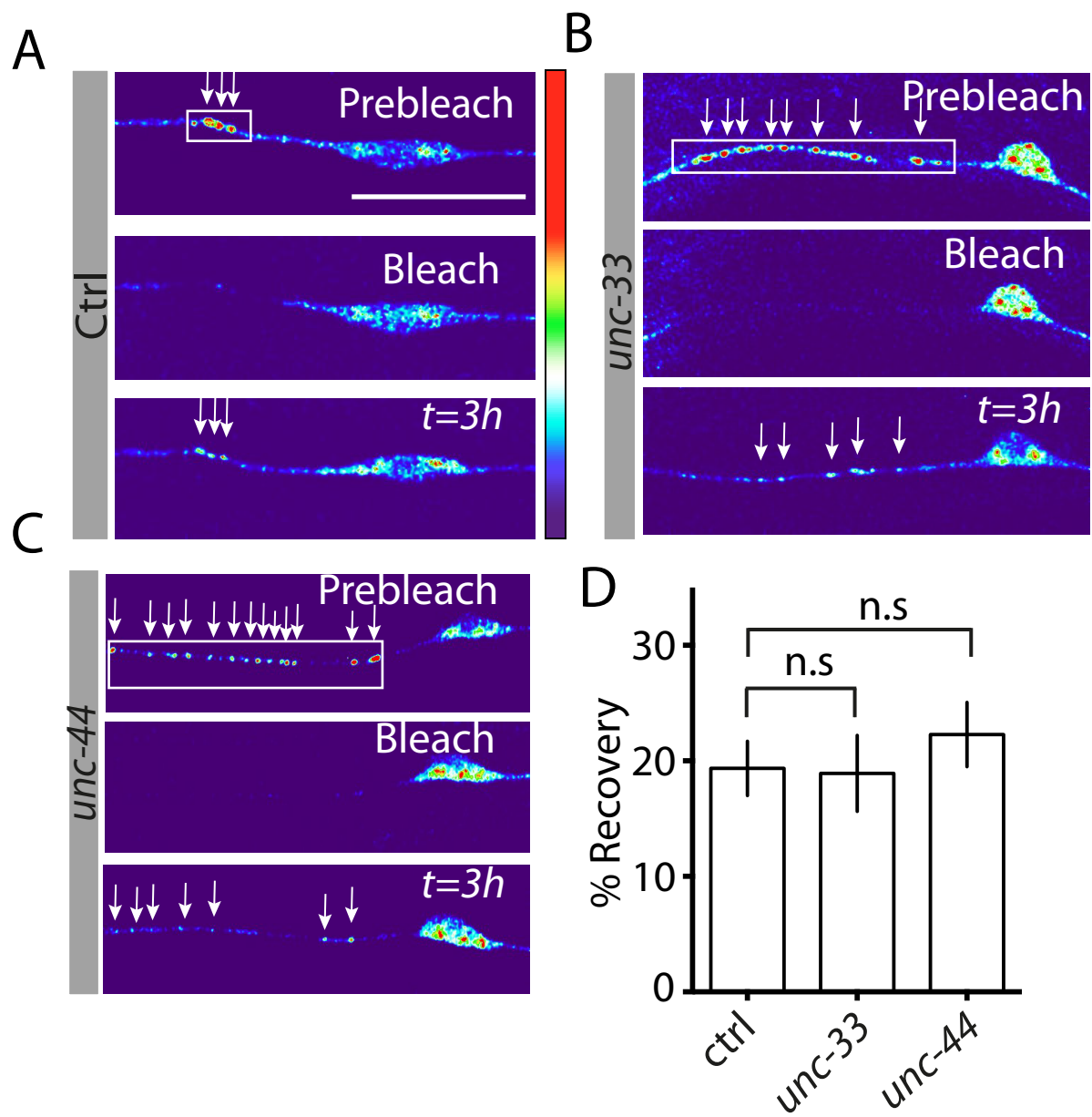

S4 Figure

Supplement: S4 Fig — Image(A) and quantification(D) show that the fluorescence intensity at zone 1 recover 20% 3 hours after photobleaching. The thermal bar shows the color code for fluorescence intensity from purple (low) to red (high). Images(B, C) and quantification(D) show that loss of function of unc-33 or unc-44 doesn’t affect the recovery after photobleaching. N>10 animals for each experiments. Data are shown as mean ± SD. ns: no significant difference. Scale bar: 10 μm. (PDF) [file pgen.1005948.s005.pdf]

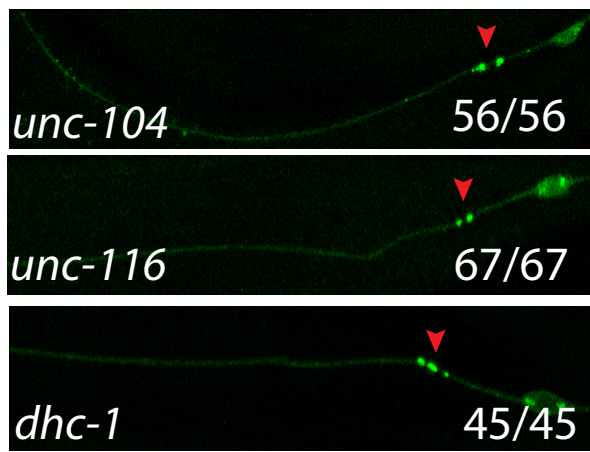

Supplement: S5 Fig — Loss of function of neuronal kinesin unc-104 and unc-116, and dynein heavy chain dhc-1 does not affect UNC-9 plaques. The number displayed at each images shows the number of animals with wild type GFP::UNC-9 plaques/ total animals. Scale bar: 10 μm. (PDF) [file pgen.1005948.s006.pdf]
